# Deciphering Host–Pathogen Interactions: Role of *Cryptosporidium* in Tumorigenesis

**DOI:** 10.3390/pathogens14030208

**Published:** 2025-02-20

**Authors:** Shakeel Hussain, Qurrat ul Ain, Muhammad Aamir, Khalid M. Alsyaad, Ahmed Ezzat Ahmed, Jude G. Zakai, Haytham Ahmed Zakai, Yongzhong Hou

**Affiliations:** 1 School of Life Sciences, Jiangsu University, Zhenjiang 212013, China; shakil_alhusaini@hotmail.com; 2 School Education Department, Government of Punjab, Mailsi 61200, Pakistan; aeniaslam38@gmail.com; 3 Chemical Engineering Department, School of Sciences, Harbin Institute of Technology, Shenzhen 518000, China; haamir99@stu.hit.edu.cn; 4 Department of Biology, College of Science, King Khalid University, P.O. Box 9004, Abha 61413, Saudi Arabia; alsyaad@kku.edu.sa (K.M.A.); aabdelrahman@kku.edu.sa (A.E.A.); 5 Prince Sultan Bin Abdelaziz for Environmental Research and Natural Resources Sustainability Center, King Khalid University, Abha 61421, Saudi Arabia; 6 Faculty of Medicine, King Abdulaziz University, Jeddah 21589, Saudi Arabia; jzakai@stu.kau.edu.sa; 7 Medical Laboratory Sciences Department, Faculty of Applied Medical Sciences, King Abdulaziz University, Jeddah 21589, Saudi Arabia

**Keywords:** *Cryptosporidium*, inflammation, cancer pathways, apoptosis, immune microenvironment, tumorigenesis

## Abstract

*Cryptosporidium*, a protozoan parasite affecting the gastrointestinal system, is primarily known for causing diarrhea, especially in those with weakened immune systems. However, there is increasingly persuasive evidence that it may be directly involved in tumorigenesis. This review examines some of the potential mechanisms through which *Cryptosporidium* infections can induce cancer, specifically chronic inflammation, manipulation of the immune system, and alteration of cell signaling pathways. Persistent inflammation with immune system changes due to chronic infection, particularly among immunocompromised hosts, leads to a microenvironment that facilitates tumorigenesis. *Cryptosporidium* manipulates important cellular pathways such as PI3K, NF-κB, Wnt, and p38/MAPK to promote cell survival, regulate immune responses, and foster tissue remodeling, all of which contribute to a tumor-friendly microenvironment. Moreover, *Cryptosporidium* virulence factors such as ROP1, sPLA2, and microRNAs disrupt host cellular stability and significantly alter host cellular gene expression, which also exacerbates inflammation and tissue damage. Epidemiological data have indicated higher rates of *Cryptosporidium* infection in cancer patients, especially patients with gastrointestinal cancers. This, among other observations, raises the possibility that the infection may be connected to cancer progression. In animal models, especially studies with *C. parvum*-challenged rodents, chronic inflammation, immune repression, and genetic mutations related to neoplasia have been reported. While this has provided us with valuable information, we still have a long way to go to fully understand the long-term ramifications of *Cryptosporidium* infection. These cover aspects such as the contribution of latent infections and the genetic diversity of *Cryptosporidium* strains in cancer. Further investigation is urgently needed to understand the molecular processes by which *Cryptosporidium* might contribute to carcinogenesis and explore potential strategies for therapy and prevention especially among immunocompromised populations.

## 1. Introduction

**Figure 1:**
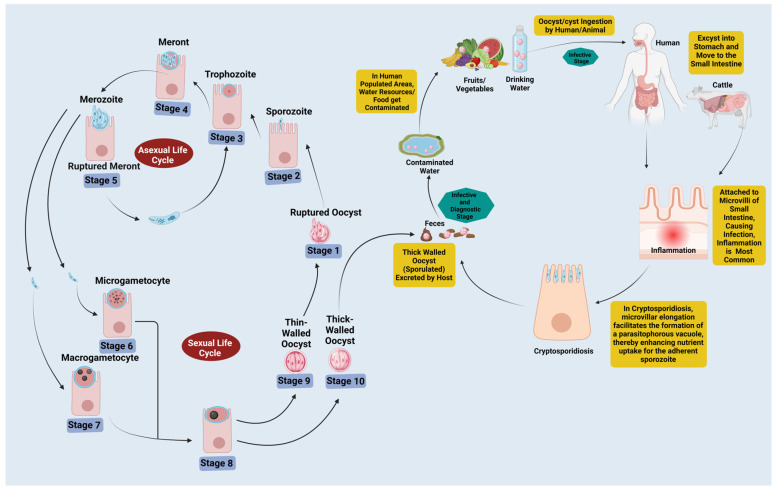
**Life cycle and transmission dynamics of *Cryptosporidium***: Infected individuals shed thick-walled oocysts in their feces, contaminating food and water. Ingestion of these leads to infection as the oocysts release sporozoites (Stages 1–2), which invade intestinal cells. Inside, they develop into trophozoites (Stage 3) that reproduce asexually to form type I meronts (Stage 4), and the type I meronts releasing merozoites (Stages 5–6). This triggers the sexual phase, forming microgamonts and macrogamonts (Stages 7–8). Their fusion creates diploid zygotes (Stage 9), which undergo meiosis and sporogony to produce oocysts (Stage 10). Thick-walled oocysts exit the body, spreading infection, while thin-walled oocysts can cause reinfection within the host (autoinfection). Created with BioRender.com.

*Cryptosporidium* is a significant protozoan parasite affecting humans, animals, and wildlife. It ranks as one of the top causes of moderate-to-severe diarrhea in young children, particularly in developing countries, and is the fourth most prevalent globally [1,2,3]. This diarrheal disease is called cryptosporidiosis [4]. *Cryptosporidium* undergoes a complex life cycle (both asexual and sexual stages) within a single host [5], ultimately resulting in the formation of oocysts that possess resistance to the environment [6]. Following ingestion, four contagious sporozoites are released from each oocyst. Subsequently, the sporozoites adhere to the apical surface of intestinal epithelial cells before penetrating the host cell membrane. This penetration results in the formation of an internal but extracytoplasmic parasitophorous vacuole (PV) [7]. The sporozoites subsequently evolve into trophozoites within the vacuole, where they reproduce three times asexually and once sexually. This process generates thin-walled or thick-walled oocysts, which contain four haploid sporozoites [7,8]. The thick-walled oocysts, which are resistant to the environment, have two layers of membranes. They are excreted in feces and infect susceptible hosts rapidly. In contrast, the thin-walled oocysts rupture in the intestinal lumen, releasing naked contagious sporozoites that infect neighboring enteric cells, thereby sustaining the infection (Figure 1). Cases of cryptosporidiosis have been reported from over 90 countries spanning all continents except Antarctica [3]. Different species of *Cryptosporidium* have been discovered worldwide, the two most widespread being *C. hominis* and *C. parvum* which can be found across Europe, the Americas, Australia and Africa [9,10]. At present, 44 species of *Cryptosporidium* have been documented, and genetic analysis has identified approximately 120 distinct genotypes [11]. Humans are typically infected by *C. hominis* and *C. parvum* [9,10], suggesting a correlation between their respective species and an array of clinical symptoms they bring on. Studies are providing increasing evidence that chronic *Cryptosporidium* infections could play a part in cancer development in immunocompromised people. This can take place through mechanisms including inflammation, immune system modulation, cell disruption, and genetic alteration [12,13,14,15,16,17,18,19]. *Cryptosporidium* manipulates several host cell signaling pathways, including PI3K, Src, Cdc42, Rho GTPases, NF-κB, and Wnt. It enhances cell survival, modulates immune responses, remodels the cytoskeleton, and potentially promotes oncogenesis in tissues affected by prolonged infection [20,21,22,23,24,25,26,27,28].

*Cryptosporidium* prevents apoptosis and promotes cell survival, possibly encouraging tumor development. NF-κB activation boosts anti-apoptotic protein and inhibits pro-apoptotic factors. Thus, caspase activation is prevented, allowing *Cryptosporidium* to maintain chronic infection and escape immune detection [23,29,30,31,32,33,34,35]. Further tumor support is provided by *Cryptosporidium*-induced inflammation. This happens by increasing cytokines including IL-6, IL-10, and TGF-β. This causes inflammation and helps *Cryptosporidium* avoid immune responses. Immune checkpoints are activated, and MDSCs and TAMs are attracted to the infection site to aid immune evasion [36,37,38,39]. Beyond these immune-modulating effects, *Cryptosporidium* alters host cell adhesion molecules, weakens immune responses, and influences chemokine production. These changes enable a persistent infection, hinder immune clearance, and increase its ability to avoid the host’s immune system, thus fostering a tumorigenic environment [24,40,41,42,43,44,45,46].

Additionally, *Cryptosporidium* utilizes virulence factors such as ROP1, sPLA2, and miRNAs to disrupt host cell integrity and immune responses. These factors disrupt cellular functions and modify the host’s transcriptomic profiles, influencing cancer-related pathways such as Hedgehog, Wnt, and p38/MAPK. This leads to chronic inflammation, tissue damage, and may create conditions favorable for carcinogenesis [40,47,48,49,50,51,52]. Rodent models have provided evidence of the involvement of *Cryptosporidium parvum* modulation, and genetic alterations. Key pathways such as Wnt signaling, immune suppression, and changes in the microbiota have been identified as playing a role in tumor promotion. However, several important questions remain, especially regarding the long-term consequences of infection, latent infections, and the complex interactions between the host and parasite [24,37,53,54,55]. There is an urgent need for further research to explore the molecular mechanisms behind these processes, investigate the virulence profiles of different *Cryptosporidium* strains, and assess the long-term cancer risks associated with *C. parvum* infections [25,56,57]. This review highlights the growing evidence that *Cryptosporidium* may function as an oncogenic agent by promoting chronic inflammation, evading immune responses, and disrupting key cancer-related signaling pathways. It stresses the critical need for further research into its potential role in cancer development and the formulation of preventive and therapeutic strategies, particularly for immunocompromised individuals.

## 2. Rationale for Investigating the Oncogenic Potential of *Cryptosporidium* Infection

*Cryptosporidium* is commonly associated with digestive ailments in immunocompromised individuals. Although its exact role remains unproven, recent research highlights chronic infections, inflammation, immune modulation, and cellular disruption as possible contributors to cancer. *Cryptosporidium* has the capacity to cause sustained inflammation as well as stress-inducing effects that contribute significantly to driving cancer progression [12]. *Cryptosporidium* may alter immune responses and foster oncogenesis [13]. Animal models demonstrate how co-infection with *Cryptosporidium* worsens disease outcomes by increasing cell damage which may ultimately result in cancerous growth [14]. Disruption of intestinal cell integrity increases tissue susceptibility to cancer-causing mutations [15], while its histone methyltransferase activity alters host chromatin, altering cancer pathways [16]. Chronic *Cryptosporidium* infections in immunocompromised populations such as those living with HIV/AIDS increase tissue damage, increasing cancer risks [17]. Furthermore, genomic analyses reveal pathways that interfere with host processes to increase mutation susceptibility [18]. The effect of *Cryptosporidium* on immune responses and glycoprotein expression may contribute to creating an environment conducive to cancer development [19]. Although not yet classified as an oncogenic agent, its chronic effects suggest further examination regarding its link to cancer risk.

## 3. Epidemiological Evidence Linking *Cryptosporidium* Infection to Cancer

*Cryptosporidium* infections are notably more common in cancer patients, particularly those with gastrointestinal cancers including colorectal, esophageal, liver, and small intestinal cancers [58,59,60]. Due to weakened immune systems, cancer patients are more susceptible to infections, and infection rates tend to be higher in this group [61]. *Cryptosporidium* is associated with gastrointestinal infections and may play a role in colorectal cancer development. Studies show that cancer patients, especially those with colorectal cancer, experience higher infection rates. For instance, in China, 17.24% of colorectal cancer patients tested positive for *Cryptosporidium*, a much higher rate compared to healthy controls [25]. Colorectal cancer is the cancer most frequently associated with *Cryptosporidium* infections. Studies report a higher prevalence among colorectal cancer patients, including 13% among cancer patients, compared to just 4% in the control group in Poland and 17.24% in China [25,62]. Chemotherapy further exacerbates infection rates, with *Cryptosporidium parvum* in 54% of chemotherapy patients as compared to 30% of the control group in Iraq testing positive [63]. Similarly, in Egypt, *Cryptosporidium* was detected in 32.5% of colorectal cancer patients [64]. Zoonotic subtypes like *C. parvum* have also been found in colorectal cancer patients [65]. In vitro studies demonstrate that *Cryptosporidium* infection in colorectal cancer cell lines promotes the expression of oncogenic microRNAs, further implicating the parasite in cancer development [66]. The chronic inflammation caused by *Cryptosporidium* may contribute to dysplastic changes, which are early signs of cancer development [67].

Moreover, *Cryptosporidium* has also been linked to other gastrointestinal cancers. In Lebanon, 21% of colonic neoplasia patients tested positive for the parasite [68], and in China, 14.29% of liver cancer patients and 6.25% of esophageal cancer patients tested positive for the parasite [25]. These findings highlight a broader association between *Cryptosporidium* and gastrointestinal cancers beyond colorectal malignancies. Elevated infection rates are also observed in pediatric oncology patients [69]. In Egypt, *Cryptosporidium* infections were reported in 23.8% of cancer patients, with even higher rates among immunodeficient adults and children [70]. *Cryptosporidium* has also been identified in lung cancer patients [71,72], though further studies are needed to understand the significance of this association.

Case–control studies further support the association between *Cryptosporidium* and cancer, especially in immunocompromised patients. A meta-analysis of 19 studies found that cancer patients are more than three times as likely to be infected [73]. Meta-analyses show that cancer patients are 3.3 times more likely to harbor *Cryptosporidium* than healthy individuals [62,73]. Elevated infection rates are also observed in pediatric oncology patients [74], and *Cryptosporidium* has been found in lung cancer patients as well [71,72]. In summary, elevated infection rates have been consistently observed in studies involving colorectal cancer [62], pediatric oncology [69], and chemotherapy patients [75].

## 4. Mechanisms of Oncogenesis

### 4.1. Hijacking Cellular Pathways

**Figure 2:**
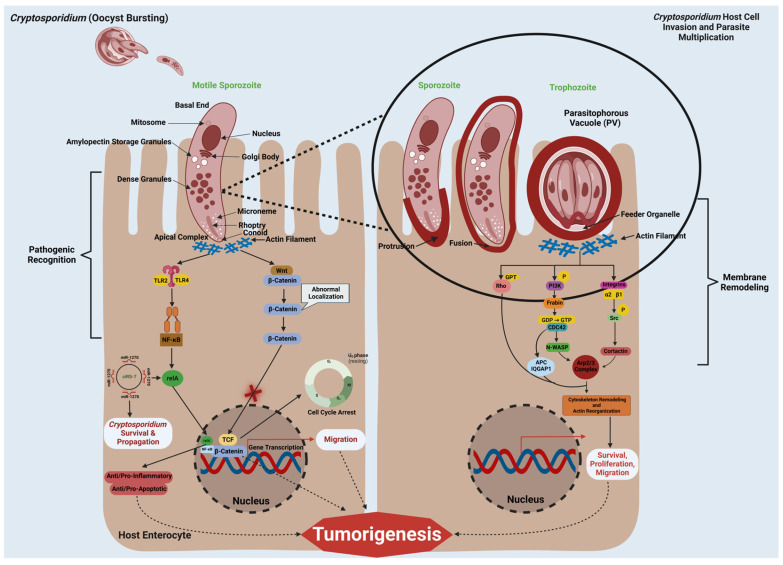
**Hijacking cellular pathways and oncogenic mechanisms in *Cryptosporidium* infections:*** Cryptosporidium* manipulates several key host cell signaling pathways to ensure its survival and potentially contribute to cancer development. Upon contact with epithelial cells, the parasite activates multiple signaling proteins, including PI3K, Src, Cdc42, and Rho GTPases. This disruption of normal cellular signaling breaks down the epithelial barrier, helping the parasite form a specialized vacuole, the parasitophorous vacuole (PV), where it can thrive. *Cryptosporidium* also triggers inflammation by activating NF-κB signaling through Toll-like receptors (TLR2/4), which not only helps the parasite avoid cell death but also promotes its replication. In addition, *Cryptosporidium* interferes with the Wnt signaling pathway, altering the location of β-catenin. Disruptions to cell cycle regulation, adhesion, and migration create conditions more conducive to tumor development. Furthermore, *Cryptosporidium* may remodel host cytoskeletons using molecules like c-Src, PI3K and Rho GTPases, making entry easier while altering host cell structures that may promote cancer progression. Host microRNAs like miR-27b and ciRS7 further regulate immune responses and gene expression that foster oncogenesis. ITGA2 and ITGB1 play pivotal roles in signal transduction that enhance the migration/survival of host cell populations, thus encouraging tumorigenic processes that might support tumorigenic processes. Created with BioRender.com.

*Cryptosporidium* uses host cell signaling pathways to support its survival and replication (Figure 2). It activates pathways like PI3K, Src, Cdc42, and Rho GTPases upon contacting epithelial cells, disrupting the cell barrier and promoting actin filament polymerization. This enables the formation of the parasitophorous vacuole (PV), essential for its survival [20,21,23,76]. Additionally, *Cryptosporidium* also triggers NF-κB signaling via Toll-like receptors (TLR2 and TLR4), inducing inflammatory cytokines and anti-apoptotic proteins to prevent host cell death and sustain replication [22,77]. Moreover, manipulation of Wnt signaling pathway impairs β-catenin localization and interferes with cell cycle regulation and adhesion, potentially contributing to oncogenesis [24]. Host miRNAs like miR-27b and ciRS-7 may also change during infection by impacting both NF-kB pathway activity as well as immune responses. These modifications support parasite survival by altering gene expression regulation [26,78], while at the same time encouraging tumorigenesis via gene regulation mechanisms that influence gene regulation [26,78].

Cytoskeletal remodeling is key in *Cryptosporidium* infection and could even contribute to oncogenesis. *Cryptosporidium* triggers activation of c-Src and PI3K at the host–parasite interface, driving actin polymerization that supports parasitophorous vacuole formation and host cell entry [21,76]. Arp2/3 complex and cortactin are also essential in acting on actin remodeling and transport of vesicles within cells, altering host cell structure to promote tumorigenesis [21,76]. Furthermore, Rho GTPases such as Cdc42 and RhoA are essential regulators of cytoskeletal dynamics, migration, survival, as well as gene expression related to cancer behaviors [21,27,76,79]. Additionally, Integrins such as ITGA2 and ITGB1 enhance host cell migration and survival to increase oncogenic potential [28].

### 4.2. Resistance to Apoptosis

**Figure 3:**
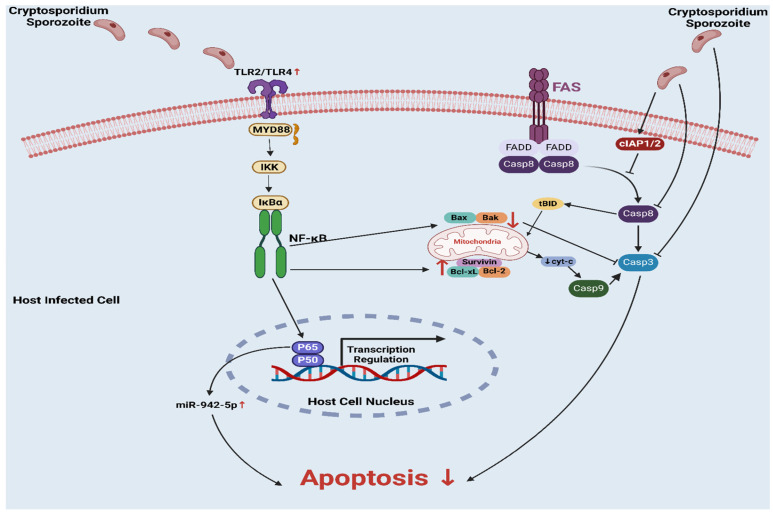
**Mechanisms of apoptosis resistance in *Cryptosporidium* infections:*** Cryptosporidium* survives longer and may cause cancer by preventing apoptosis in infected cells. It activates NF-κB signaling as a crucial mechanism, which boosts cell-death-preventing proteins such Bcl-2, Bcl-xL, and survivin. *Cryptosporidium* keeps infected cells alive by upregulating anti-apoptotic proteins. The parasite also inhibits caspases and pro-apoptotic genes like Bax and Bak. Apoptosis resistance may cause cancer. *Cryptosporidium* also manipulates apoptosis inhibitors like cIAP1 and cIAP2 to help infected cells survive and become malignant. Created with BioRender.com.

One key way that *Cryptosporidium* contributes to tumorigenesis is its ability to suppress apoptosis among infected cells through the activation of NF-kB signaling pathways (Figure 3). NF-kB enhances the expression of anti-apoptotic proteins that protect infected cells from programmed cell death and support their survival when exposed to host immune reactions [23,29,30]. Resistance to apoptosis creates an environment in which infected cells persist longer, giving rise to mutations that could drive oncogenesis. *Cryptosporidium* also blocks key apoptotic mediators such as caspase-3 and caspase-8 that regulate DNA fragmentation and cell breakdown. By keeping these caspases active for as long as possible, *Cryptosporidium* ensures cell survival while prolonging infection [31].

*Cryptosporidium* exerts control over both pro-apoptotic and anti-apoptotic gene expression, helping sustain cell survival during infection. For example, anti-apoptotic genes like Bcl-2, Bcl-xL and survivin become upregulated upon infection. These proteins stabilize mitochondrial membranes and prevent cytochrome-c release which would otherwise trigger activation of the apoptotic pathway [32,33]. In contrast, *Cryptosporidium* effectively suppresses pro-apoptotic genes such as Bax and Bak, thus avoiding mitochondrial membrane permeabilization and subsequent cell death, aiding its ability to avoid immune responses while remaining chronic [34,35]. Furthermore, *Cryptosporidium* modulates expression levels of two anti-apoptotic regulators known as cellular inhibitors of apoptosis (cIAP1 and cIAP2), blocking caspase activity to extend cell survival, thus increasing tumorigenic potential [35].

### 4.3. Inflammatory Response and Tumor Microenvironment

**Figure 4:**
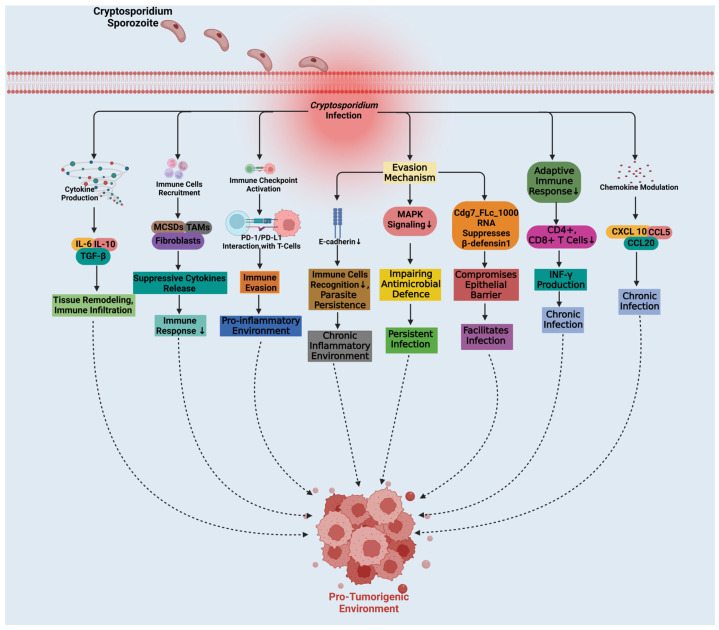
**Inflammatory responses and tumor microenvironment in *Cryptosporidium* infections:*** Cryptosporidium* infection causes chronic inflammation in the host, creating a tumor-promoting environment. Infected tissues release cytokines such as IL-6, IL-10, and TGF-β, promoting tissue remodeling, immune cell infiltration, and cell survival. *Cryptosporidium* also recruits immune cells like MDSCs and TAMs to assist tumor growth and escape the immune system. To avoid immunological identification, *Cryptosporidium* modifies cell surface proteins such as E-cadherin and immune checkpoints like PD-1/PD-L1. By suppressing MAPK signaling, reducing the activity of CD4+ and CD8+ T-cells, and drawing in regulatory immune cells, *Cryptosporidium* is able to maintain chronic infection and create an environment that suppresses immune responses, further enhancing its potential to drive tumorigenesis. *Cryptosporidium* also regulates chemokines in ways that support immune evasion and intensify the inflammation that promotes tumor growth. Created with BioRender.com.

Chronic inflammation caused by *Cryptosporidium* infections plays a crucial role in creating an environment conducive to tumor development (Figure 4). Persistent inflammation results in the release of pro-tumorigenic cytokines such as IL-6, IL-10 and TGF-b that drive tissue remodeling and immune cell infiltration and create an environment favorable to tumorigenic growth [36,37]. Furthermore, these cytokines promote tumor development through increasing cell survival, proliferation, and immune evasion, enhancing tumor development further [36,37]. Immunosuppressed hosts exposed to *Cryptosporidium* have shown elevated Cyclin D1 expression, an important cell cycle regulator associated with intestinal dysplasia and precancerous changes. This link indicates a correlation between inflammation, epithelial damage, and tumorigenesis [38]. Also, inflammation attracts myeloid-derived suppressor cells (MDSCs), tumor-associated macrophages (TAMs) and fibroblasts (cells known to release additional cytokines that suppress immunity and support tumor progression) [37]. *Cryptosporidium* activates immune checkpoints such as PD-1/PD-L1 that enable immune evasion while simultaneously creating an environment conducive to tumor development [39].

*Cryptosporidium* employs several immune evasion strategies to aid tumorigenesis. For instance, they modulate E-cadherin expression to decrease immune recognition of infected cells, prolonging chronic inflammation [24]. Moreover, the parasite suppresses MAPK signaling in intestinal epithelial cells, weakening antimicrobial defenses and allowing infection to persist [40]. *Cryptosporidium* also delivers RNA transcripts like Cdg7_FLc_1000, which downregulate beta-defensin 1 (DEFB1), compromising the epithelial barrier and promoting infection [41]. These actions create an immunosuppressive environment conducive to sustained infection as well as tumorigenesis.

*Cryptosporidium* suppresses adaptive immunity by targeting CD4+ and CD8+ T-cells and decreasing interferon-gamma production, leading to chronic infection as well as creating an environment conducive to tumorigenesis [42]. Additionally, recruitment of regulatory immune cells further inhibits effector T-cell functions, creating an immunosuppressive tumor microenvironment [43]. Chemokines like CXCL10, CCL5, and CCL20 are involved in immune cell recruitment during infection. However, reduced CCL20 expression increases parasite persistence, as shown by higher parasite loads in neonatal mice. This manipulation of immune signaling promotes chronic infection and supports tumorigenesis [44,45,46,80,81].

## 5. Molecular Factors Involved in *Cryptosporidium*-Induced Carcinogenesis

### 5.1. Virulence Factors

*Cryptosporidium* uses virulence factors to invade host cells and damage tissues. Proteins like ROP1 disrupt epithelial integrity by targeting LIM domain only 7 (LMO7), aiding parasite replication [47]. Additionally, Secretory phospholipase A2 (sPLA2) breaks down host membranes, enabling invasion, with its inhibition significantly reducing parasite entry [48]. In addition to protein effectors, *Cryptosporidium* also alters host microRNAs (miRNAs) to modulate gene expression. For example, miR-221 downregulation increases ICAM-1 expression, promoting inflammation and potentially tumorigenesis [49]. Computational studies suggest that infection-triggered miRNAs affect pathways like Wnt and Hedgehog, linked to cancer progression [50]. These combined mechanisms enable *Cryptosporidium* to survive while fostering conditions that may lead to carcinogenesis in chronic infections.

### 5.2. Transcriptomic Changes

*Cryptosporidium* infection significantly alters host gene expression, particularly in intestinal epithelial cells. Transcriptomic studies show upregulation of immune response genes like IL-1β, IFN-γ, and TNF-α, which initiate inflammation and help control the parasite. However, this inflammatory response can also damage tissues, promoting chronic disease or malignancy [40]. Additionally, in *C. parvum*-infected HCT-8 cells, researchers identified over 800 differentially expressed lncRNAs and 1300 mRNAs involved in pathways affecting tight junctions, inflammation, and signaling, which are key processes for maintaining epithelial integrity during infection [50]. *Cryptosporidium* also influences cancer-related pathways like Hedgehog and p38/MAPK pathways, where improper regulation increases cell proliferation while disrupting growth control, elevating cancer risk [46]. Suppressing these pathways weakens immune defenses and disrupts epithelial homeostasis, enhancing chronic inflammation and cell stress.

*Cryptosporidium* also plays a part in cancer risk by manipulating cancer-associated pathways like Hedgehog and p38/MAPK signaling pathways, with dysregulated Hedgehog signaling leading to cell proliferation and breakdown, disrupted growth control mechanisms, increasing cancer risks [50]. Subverting the p38/MAPK pathway weakens immune defenses and disrupts epithelial homeostasis, contributing to chronic inflammation and cell stress [37,51]. *Cryptosporidium* also injects host cells with Cdg7_FLc_0990 transcripts, recruiting histone methyltransferases that epigenetically modify genes like LRP5 and IL33, leading to impaired immunity, homeostasis disruptions and oncogenic transformation [52]. Together, these molecular alterations exacerbate chronic inflammation, immune suppression, and pathway disruption, damage tissue significantly, and increase cancer susceptibility under prolonged infection [40].

## 6. Experimental Models and Future Research Directions

### 6.1. Animal Models

Rodent models have proven useful in exploring the carcinogenic potential of *Cryptosporidium parvum*, particularly among immunocompromised hosts. These studies reveal how chronic *C. parvum* infection drives tumorigenesis by creating chronic inflammation, altering DNA sequences and disrupting pathways such as Wnt signaling. These changes create a tumor microenvironment (TME) characterized by immune suppression and inflammation as well as recruitment of tumor-associated macrophages and fibroblasts [37]. In addition, genomic analyses reveal significant variation among *C. parvum* isolates when it comes to their virulence; specific mutations affecting membrane or secreted protein influence oncogenicity [53]. For example, *C. parvum* in dexamethasone-treated SCID mice disrupts the localization of β-catenin and p53 as well as adherens junctions, further supporting its role in tumor development [24]. Furthermore, novel 3D murine colonic explant models show chronic *C. parvum* infection alone can trigger cancerous lesions similar to in vivo findings [54]. Furthermore, new 3D murine colonic explant models validate that chronic *C. parvum* infection by itself can induce neoplastic lesions, proving in vivo data [82]. Chronic infections inhibit protective intraepithelial lymphocytes, including gamma delta+ T-cells, thereby promoting tumorigenesis [83]. In addition, alterations in gut microbiota during infection play a role in chronic inflammation and the risk of cancer [55]. Mouse models demonstrate the significance of *C. parvum* in carcinogenesis, emphasizing chronic inflammation, immune modulation, and genetic factors [84]. These models are invaluable for studying therapeutic strategies targeting Wnt signaling, immune suppression, and virulence markers.

### 6.2. Gaps in Current Knowledge

While significant progress has been made in understanding the oncogenic potential of *C. parvum*, critical knowledge gaps remain. Most research focuses on acute infections, leaving the long-term effects underexplored. Although rodent models show that chronic low-dose infections can cause adenocarcinomas within weeks, studies are needed to track molecular changes over extended periods, particularly in immunocompetent hosts [56]. Furthermore, the impact of latent infections is also unclear. *C. parvum* has been associated with dysplastic changes in the gastrointestinal tract, but whether these changes progress to cancer decades later remains unknown. Epidemiological studies linking past *C. parvum* infections to later cancer diagnoses could provide valuable insights into this potential delayed connection [54].

Key gaps remain in understanding the role of *C. parvum* in carcinogenesis. While pathways like Wnt signaling are implicated, the influence of host genetic predispositions and the mechanisms behind the immunosuppressive tumor microenvironment (TME) require further study [37]. Advanced tools like CRISPR and transcriptomics could uncover how *C. parvum* manipulates host immunity and drives neoplastic changes. Virulence genes, including those coding for membrane proteins, appear significant but need more investigation [53]. Moreover, genetic diversity among *C. parvum* strains, with some showing higher oncogenic potential, highlights the need for comparative studies [57]. Large-scale epidemiological research is also essential to quantify cancer risk, particularly in regions with high cryptosporidiosis rates [25]. Multidisciplinary approaches using molecular tools, animal models, and human studies are critical to advancing understanding and developing preventive measures.

## 7. Conclusions

Emerging evidence points to *Cryptosporidium* as a possible cancer-causing agent. The parasite seems to promote chronic inflammation, evade immune detection, and resist cell death, all of which could contribute to cancer development. It activates pro-survival pathways like NF-κB, boosts anti-apoptotic proteins, and influences key cancer-related pathways, such as Wnt and Hedgehog, driving cellular transformations. Additionally, *Cryptosporidium* creates a pro-inflammatory tumor microenvironment that supports immune suppression and aids in tissue remodeling, further encouraging malignancy. While these findings highlight the potential of *Cryptosporidium* in cancer development, more research is needed to understand the long-term effects of infection and how different *Cryptosporidium* strains might contribute to cancer. Delving deeper into these mechanisms could pave the way for new strategies to prevent and treat cancer linked to *Cryptosporidium* infection. By targeting the molecular pathways involved, we may be able to reduce the cancer burden, particularly in immunocompromised populations.
